# Equality in Maternal and Newborn Health: Modelling Geographic Disparities in Utilisation of Care in Five East African Countries

**DOI:** 10.1371/journal.pone.0162006

**Published:** 2016-08-25

**Authors:** Corrine W. Ruktanonchai, Nick W. Ruktanonchai, Andrea Nove, Sofia Lopes, Carla Pezzulo, Claudio Bosco, Victor A. Alegana, Clara R. Burgert, Rogers Ayiko, Andrew SEK Charles, Nkurunziza Lambert, Esther Msechu, Esther Kathini, Zoë Matthews, Andrew J. Tatem

**Affiliations:** 1 WorldPop Project, Geography & Environment, University of Southampton, Southampton, United Kingdom; 2 Flowminder Foundation, Stockholm, Sweden; 3 ICS Integrare, Barcelona, Spain; 4 The DHS Program, ICF International, Rockville, Maryland, United States of America; 5 Open Health Initiative, East African Community Secretariat, Arusha, Tanzania; 6 Ministry of Public Health and Fight Against AIDS, Bujumbura, Burundi; 7 Ministry of Health, Community Development, Gender, Elderly and Children, Dar es Salaam, Tanzania; 8 Ministry of Health, Nairobi, Kenya; 9 Division of Social Statistics and Demography & Centre for Global Health, Population, Poverty and Policy, Faculty of Social and Human Sciences, University of Southampton, Southampton, United Kingdom; Johns Hopkins Bloomberg School of Public Health, UNITED STATES

## Abstract

**Background:**

Geographic accessibility to health facilities represents a fundamental barrier to utilisation of maternal and newborn health (MNH) services, driving historically hidden spatial pockets of localized inequalities. Here, we examine utilisation of MNH care as an emergent property of accessibility, highlighting high-resolution spatial heterogeneity and sub-national inequalities in receiving care before, during, and after delivery throughout five East African countries.

**Methods:**

We calculated a geographic inaccessibility score to the nearest health facility at 300 x 300 m using a dataset of 9,314 facilities throughout Burundi, Kenya, Rwanda, Tanzania and Uganda. Using Demographic and Health Surveys data, we utilised hierarchical mixed effects logistic regression to examine the odds of: 1) skilled birth attendance, 2) receiving 4+ antenatal care visits at time of delivery, and 3) receiving a postnatal health check-up within 48 hours of delivery. We applied model results onto the accessibility surface to visualise the probabilities of obtaining MNH care at both high-resolution and sub-national levels after adjusting for live births in 2015.

**Results:**

Across all outcomes, decreasing wealth and education levels were associated with lower odds of obtaining MNH care. Increasing geographic inaccessibility scores were associated with the strongest effect in lowering odds of obtaining care observed across outcomes, with the widest disparities observed among skilled birth attendance. Specifically, for each increase in the inaccessibility score to the nearest health facility, the odds of having skilled birth attendance at delivery was reduced by over 75% (0.24; CI: 0.19–0.3), while the odds of receiving antenatal care decreased by nearly 25% (0.74; CI: 0.61–0.89) and 40% for obtaining postnatal care (0.58; CI: 0.45–0.75).

**Conclusions:**

Overall, these results suggest decreasing accessibility to the nearest health facility significantly deterred utilisation of all maternal health care services. These results demonstrate how spatial approaches can inform policy efforts and promote evidence-based decision-making, and are particularly pertinent as the world shifts into the Sustainable Goals Development era, where sub-national applications will become increasingly useful in identifying and reducing persistent inequalities.

## Introduction

Worldwide maternal deaths have been cut nearly in half over the past two and a half decades, largely due to a committed global effort to improve the lives and wellbeing of the world’s most vulnerable populations.[1] Despite substantial progress among even the most disadvantaged subgroups, pregnancy remains risky for many of the world’s women, and inequalities persist across the socioeconomic spectrum.[2] These risks are borne unequally both between and within countries, with pockets of relatively remote women in rural and poor communities bearing a disproportionate burden of morbidity and mortality.[3] Historically, however, MNH policy has largely relied on aggregate, national-level statistics, which often mask these underlying spatial pockets of sub-national inequalities.[4] The United Nations therefore recently announced new Sustainable Development Goals (SDGs) to reduce these disparities and promote health and well-being for all, with specific targets to improve maternal and newborn health (MNH) outcomes among all women and children alike, and a particular emphasis on sub-national monitoring.[5] As achieving these SDG targets will necessarily require effective use of limited resources, academics and policymakers alike have begun encouraging spatial disaggregation of health data.[6] Advances in computational geostatistical techniques and the increasing availability of geo-located data have increased the ability to produce these fine spatial resolution maps, critical for uncovering historically overlooked disparities. By monitoring health within a spatial framework, policy makers can better focus resources and intervention efforts amongst the most disadvantaged and marginalized populations, ensuring advancement of SDG targets in reducing inequalities among all.[7]

Ultimately, achieving these targets and accelerating progress towards reducing adverse MNH outcomes requires understanding use of pregnancy-related services before, during, and after childbirth, in addition to understanding the underlying disparities that drive observed utilisation patterns.[8] Specifically, the World Health Organisation has identified several critical determinants in reducing preventable pregnancy-related morbidity and mortality as: 1) access to antenatal care during pregnancy, 2) skilled birth attendance during delivery, and 3) postnatal care in the days and weeks following birth.[3] Care seeking behaviour throughout the duration of a woman’s pregnancy, however, is a complicated decision fraught with delays and barriers, including individual beliefs, societal norms, monetary barriers, and geographic access to necessary services.[9]

Several studies have identified geographic accessibility specifically as a fundamental barrier in obtaining MNH care among developing nations, driving persistently high maternal and neonatal mortality rates.[10,11] This physical accessibility is driven by various geographic factors such as distance to the nearest health facility, topography of the local landscape, household transportation capacity, and road network infrastructure, all synergistically determining the extent and duration to which a woman can seek and obtain care.[12,13] Studies have therefore called for a nuanced understanding of accessibility as central to assessing maternal and newborn health and guiding policy interventions.[9–11,14]

The use of spatial statistics has become an increasingly recognised methodology to identify these hidden gaps, as geographic and spatial dynamics largely drive physical accessibility. These techniques can be used to extend an understanding of accessibility to produce policy relevant, high-resolution maps that can be used to focus resources where pregnancy is often riskiest.[10] Despite this, current spatial analyses of maternal and newborn health remain limited, and spatially explicit data currently available (such as geo-referenced Demographic and Health Surveys (DHS) and Service Provision Assessment (SPA) surveys) remain underutilised.[15,16] While previous studies have examined the impact of geographic accessibility on a handful of MNH outcomes, few studies have explored accessibility as a determinant across the spectrum of pregnancy, particularly at a disaggregated level.

Here, we examined several MNH outcomes in the context of geographic accessibility to map the likelihood of receiving care before, during, and after delivery. Specifically, we utilised a geo-referenced dataset of nearly 10,000 health facilities to generate high-resolution surfaces and administratively relevant maps reflecting the probability of a woman obtaining critical MNH care throughout the five East African Community partner states. By mapping disaggregated MNH outcomes as a function of geographic accessibility, outputs of these analyses have important policy implications in focusing future intervention efforts and allocation of resources, as well as providing a baseline from which to monitor future progress in the SDG era.

## Methods

### Overview

In this study, we modelled the probability of a woman receiving 4+ antenatal care (ANC) visits before delivery, skilled birth attendance (SBA) during delivery, and receiving postnatal care (PNC) within 48 hours of delivery, using Demographic and Health Surveys (DHS) data throughout Burundi, Kenya, Rwanda, Tanzania and Uganda. We visualised these probabilities at both high-resolution (300 x 300 m grid cells) and policy relevant (administrative level 2, typically districts) scales to highlight spatial heterogeneity and sub-national inequalities in MNH care utilisation throughout the region of interest. Because of the importance of physical accessibility for care utilisation, our primary explanatory variable of interest was geographic inaccessibility to the nearest health facility.

To explore MNH utilisation in the context of geographic inaccessibility, we created a gridded travel impedance surface reflecting overall ease of traversing each 300 x 300 m square in the study region, given the topography and transport infrastructure of the region. We subsequently used this impedance surface to inform a cost-distance analysis, generating a geographic inaccessibility score for each 300 m grid cell. These scores ranged from 0 (highly accessible) to 7 (highly inaccessible) and represented ease of access to the nearest health facility among a dataset of 9,314 facilities. We included this inaccessibility score as a covariate in a hierarchical mixed effects logistic regression model by overlaying DHS cluster locations on this generated accessibility surface and extracting scores for each cluster location. Finally, we used the resulting model of best fit to visualise probabilities of obtaining MNH care for each 300 x 300 m square throughout the region. Random effects included in the model consisted of DHS regions (N = 61) within the five countries, while fixed covariates included cluster-level (N = 3,311) effects of accessibility and rurality, and individual-level (N = 25,325) effects of wealth, education, and an interaction between age and total children delivered.

### Data

#### Assembling individual-level MNH data

To explore variations in utilisation of MNH care across central East Africa, we obtained data from the most recent standard DHS for each country: Kenya (2014), Tanzania (2010), Uganda (2011), Rwanda (2010), and Burundi (2010). [17–21] Data were combined and processed using SAS v. 9.4 software, [22] with a total of 72,952 respondents between the five countries. For these analyses, we included women with a birth in the preceding five years, resulting in a total of 36,460 women. We obtained corresponding GPS coordinates for cluster locations via the DHS (for detailed methods, see http://dhsprogram.com/What-We-Do/GPS-Data-Collection.cfm) and mapped these using ArcGIS 10.2.2 software.[23] To maintain participant confidentiality, the DHS randomly displaces GPS locations, with displacement diameters varying by urban (up to 2 km) and rural (up to 5 km, with 1% up to 10km) location.[24] To minimize displacement bias, we therefore drew corresponding buffers (circles placed around point locations with a specified diameter) of 2 km and 5 km around cluster locations to be used in later analyses, according to DHS guidelines.[25] Only women with associated cluster locations were consequently included in further analyses, with 36,178 women among 3,311 clusters (S1 Fig). Finally, to allow for subsequent model validation, we trained the model using 70% of the total sample with the remaining 30% set aside for model validation, resulting in 25,325 women used in the final model (see S1 File for more detail).

The University of Southampton Ethics and Research Governance approved secondary analysis of these data (ethics approval number 16918). Survey data used in these analyses are freely available via the DHS website, and participant confidentiality is outlined further at http://dhsprogram.com/What-We-Do/Protecting-the-Privacy-of-DHS-Survey-Respondents.cfm.

#### Assembling a database of health facilities

**Fig 1:**
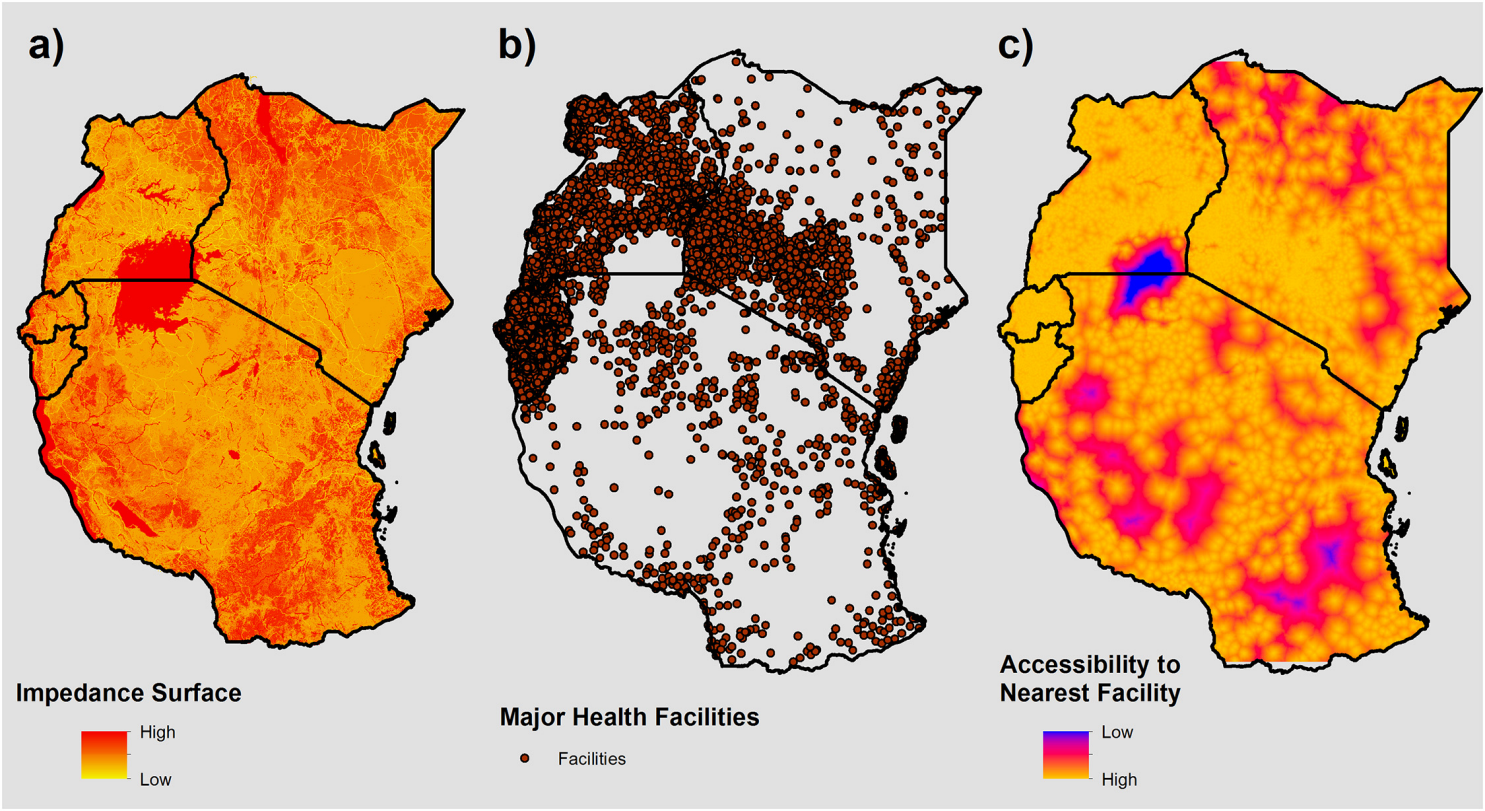
Mapping inaccessibility to the nearest health facility. **A)** Impedance surface representing the difficulty in traversing a given 300 x 300 m cell. **B)** Health facility locations used as destinations. **C)** Accessibility surface generated via cost-distance analysis using inputs **A)** and **B)**, representing the least accumulative geographic “cost” value for a 300 x 300 m cell in accessing the nearest health facility.

With input and collaboration from the intergovernmental East African Community (EAC) Secretariat, we obtained health facility data from ministries of health on over 19,000 mapped facility locations throughout the five EAC partner countries. These data included information for each health facility on operational status, private/public ownership, and type, and initially included dispensaries, maternity homes, district hospitals, health centres, and health posts. In the presented analyses, we included national, district, or regional hospitals, maternity homes, and health centres, and excluded dispensary facilities, as these facilities do not reliably offer comprehensive MNH care, including inpatient care critical for skilled birth attendance and postnatal care.[26] After excluding dispensary facilities and cleaning the dataset to correct for duplicate entries or incorrect coordinates, we used a remaining 9,314 facilities in our cost-distance analyses (see S2 File). Finally, we imported this final list of health facilities into ArcGIS software and geo-located facility locations using latitude and longitude coordinates within the dataset to create a shapefile of health facility locations throughout the study countries (Fig 1b).

### Creating an impedance surface and inaccessibility score

We combined these health facilities with a gridded impedance surface representing difficulty of travel through a given cell to generate a geographic accessibility surface that reflected the most efficient, or least “cost” accumulative, pathway to the nearest health facility. We created this impedance surface for the study region by combining data on primary, secondary and tertiary road networks, permanent water bodies and river networks via DIVA-GIS (freely available at www.diva-gis.org), land cover data from the European Space Agency’s 2009 GlobCover initiative, and elevation data from the Advanced Spaceborn Thermal Emission and Reflection Radiometer-Global Digital Elevation Model (ASTER-GDEM) Version 2.[27,28] These surfaces were rasterized, or gridded, and consolidated to create a single surface where travel speeds could be assigned to each cell. Because consolidation of these surfaces necessitates identical cell size, we aggregated data to the resolution of the coarsest surface, thereby driving a 300 x 300 m resolution throughout further analysis.

To assign travel speeds to each 300 m cell, we built upon methods outlined previously by Alegana et al. 2012.[29] While previous studies have modelled likely mode of transport used to access health facilities using sparse survey data and small area estimation approaches, [11] similar data were not available for all study countries used in these analyses. We therefore assumed mechanised transport on primary and secondary road networks, walking for off-road and tertiary networks, and that major water bodies were not traversable. For primary and secondary road networks, we assigned driving speeds of 80 and 60 km/hour, respectively. While maximum travel speeds for all five countries vary considerably, these speeds have been used in the literature previously, and were conservative estimates among the five countries.[29,30] For cells which did not contain road networks, we classified land cover into broad categories such as tree, shrub or herbaceous cover, water body, and cultivated/managed or bare area. We then assigned these categories associated walking speeds, ranging from 2 km/hour (for desert area) to 5 km/hour (for cultivated or built areas). We inferred slope from elevation data using the Slope tool in ArcGIS software, and incorporated this into walking speeds based on Tobler’s equation, which adjusts for increased walking speed on down-slopes and decreased walking speed on up-slopes.[31] Permanent major rivers and other large water bodies represented a barrier to movement in these analyses, and were therefore designated a walking speed of 0 km/hour. For a detailed list of land cover classification and associated travel speeds, see Alegana *et al*. 2012.[29] Finally, these discrete travel speed definitions were used to create standardized, ranked ‘impedance’ indices across the study region ranging from 1 to 7, where 1 represented the fastest travel speed (80 km/hr) and 7 represented the slowest speed (0 km/hr). The resulting impedance surface was then used in the cost-distance analyses (Fig 1a).

Using this impedance surface, we created an overall inaccessibility score by performing a least accumulative cost-distance analysis using the Cost Distance tool in ArcGIS software. Briefly, this tool calculates the total geographic "cost"—a relative estimate of accessibility to a “source”, in this case health facilities—for each 300 m square throughout the study region, given an input impedance surface. Each square's inaccessibility score, ranging from 0 (highly accessible) to 7 (highly inaccessible), represents the sum of the impedance values of traversed cells when traveling from that square to the facility with the lowest overall travel “cost”. Therefore, higher scores on this index represent greater geographic “cost” and greater difficulty in reaching the nearest health facility. For a more detailed description of cost distance analysis and the associated inputs, see Environmental Systems Research Institute (ESRI), 2007.[32]

Fig 1 outlines the inputs and output of this analysis, representing a) the impedance surface as described above, b) facility locations, and c) the resulting accessibility surface. Using this accessibility surface, we subsequently overlaid buffered DHS cluster locations, and extracted the mean score throughout the buffers to include as an explanatory variable in exploring probabilities of obtaining MNH care.

### Analysis of MNH outcomes

#### Hierarchical mixed effects modelling of MNH indicators

**Fig 2:**
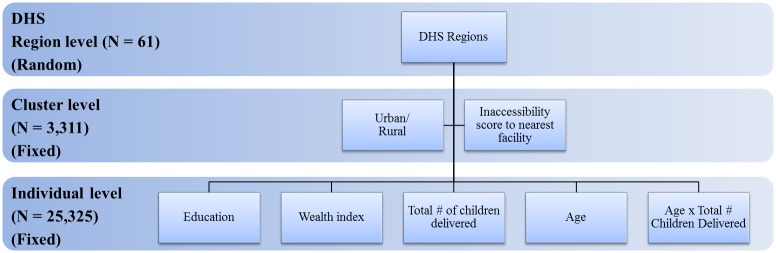
Hierarchical covariates included in the mixed-effects logistic regression analysis. Covariates are listed in boxes with corresponding hierarchy and fixed versus random effect #.

We used the inaccessibility score associated with DHS cluster locations to model the MNH outcomes of interest in the DHS data. The primary outcomes examined in these analyses included skilled birth attendance, antenatal care, and postnatal care, chosen through feedback from policy makers at the EAC organisation based on their programmatic relevance and impact. We modelled these outcomes using hierarchical mixed effects logistic regression using the ‘lme4’ package in R software.[33] Such multilevel analyses have been used previously in the literature with DHS data to account for the inherent nesting structure and multistage sampling design of the data.[34,35]. Fig 2 outlines the three hierarchical levels existing in these analyses: the individual level, the cluster level, and the regional level. By using hierarchical, mixed effects-based model inference, spatial variation as a result of region-specific contextual factors (such as road infrastructure and health financing) can be captured in the model which otherwise would have been unaccounted for, and the inherent nested sampling structure employed through the DHS may be controlled for, regardless of significance of the random effect itself.[34,36]

In these analyses, we defined skilled birth attendance as assistance during delivery by any doctor, nurse, or midwife (as defined by country-specific DHS observations), while antenatal care was defined based on WHO guidelines as 4+ antenatal visits during pregnancy, and postnatal care was defined as the respondent’s first check-up occurring within 48 hours of delivery. For ANC and PNC, the DHS captures information on the most recent live birth, while SBA is recorded among all births in the preceding 5 years. Therefore, ANC and PNC represent care received for the most recent live birth a woman had, while SBA represents the proportion of all births a woman had in the preceding 5 years which had a skilled attendant present. By examining SBA among all births in the preceding 5 years, we included all available data for the 37,309 total births occurring among the 25,325 women used in these analyses. Fig 2 outlines specific covariates used in the models, with individual level fixed effect covariates including DHS wealth quintile, highest level of education obtained, and an interaction between number of children delivered and respondent age. Lastly, DHS regions (N = 61) within the five countries were included in the model as a random effect to account for unexplained spatial variation and region-specific differences.

#### High-resolution mapping of MNH care utilization

We applied the results of the best fit model across the previously described accessibility surface to visualise the probability of a given birth receiving MNH care for each 300 x 300 m grid square. However, underlying population distributions and human settlements are highly heterogeneous and therefore variable in birth rates, limiting direct applicability of these probabilities. Therefore, to present a more accurate reflection of actual births at risk of not receiving these critical MNH services, we sought to incorporate information on the current number of live births occurring sub-nationally for the year 2015, reflecting probability of a given birth obtaining MNH care in a more policy-relevant framework.

#### Estimating births-adjusted MNH care utilisation sub-nationally

The distribution of live births in the study region was obtained via the WorldPop project at a 100 m spatial resolution, freely available at www.worldpop.org (see S2 Fig).[37] Briefly, these data incorporate population demographics and satellite imagery data such as settlements, land cover, night-time lights, and sub-national age structure to model the distribution of women of childbearing age. Sub-national age-specific fertility rates, UN population projections, and estimates of abortions, stillbirths, and miscarriages are then used to model live births and pregnancies. Detailed methodology is outlined further in Tatem et al. 2014.[38]

Using ArcGIS software, we adjusted the birth surfaces from 100 m to 300 m spatial resolution to match the resolution of the probability surface, multiplied the births and probability surfaces, and summed these values to the administrative unit level 2 to reflect actual number of births at-risk for each outcome. We then calculated the births-adjusted probability for each administrative level 2 unit throughout the region by dividing these at-risk births with total births in the administrative unit, as obtained from WorldPop. By incorporating data on live births, we present maps reflecting actual fertility rates, accounting for age structure, urban/rural differences, stillbirths, miscarriages, etc.

## Results

### Gridded accessibility surface

Fig 1c presents results of the cost-distance analysis, and represents geographic accessibility to the nearest health facility for a given 300 x 300 m square. Unsurprisingly, health facility distribution predominantly drives patterns in the accessibility surface, with remote areas lacking facilities representing the most inaccessible throughout the region, regardless of landscape topography. This result visually highlights the spatial patterns in accessibility emerging from the heterogeneous placement of major health facilities. Of note, the area of lowest accessibility observed in northern Tanzania and southern Uganda is a result of Lake Victoria, represented by red in Fig 1a. However, because neither health facilities nor DHS data are located in this area, results remain unaffected by this artefact.

### Explanatory variables of MNH care utilisation

Table 1 presents modelled odds ratios and 95% confidence intervals for each MNH outcome. Across all outcomes, increasing wealth and education was associated with greater odds of obtaining MNH care, with the most disparities between groups observed among skilled birth attendance. Specifically, those in the highest wealth quintile had over 4 times odds of delivering with a SBA as compared to those in the lowest wealth quintile (4.71; CI: 4.22–5.25), while those with higher education had over 7 times the odds of having a SBA present at delivery (7.63; CI: 5.91–9.84). In contrast, those in the richest quintile had only a 1.5 to 2 times increased odds of obtaining ANC (1.52; CI: 1.36–1.70) and PNC (1.89; CI: 1.67–2.14) as compared to those in the poorest quintile, while those with the highest education had a 2 to 3 times increased odds of obtaining ANC (2.82; CI: 2.35–3.39) and PNC (1.92; CI: 1.61–2.28) as compared to those with no education.

**Table 1:** Hierarchical mixed effects logistic regression model odds ratios of MNH outcomes among female DHS respondents in five East African countries (N = 25,325).

	**SBA**	**ANC**	**PNC**
**Fixed Effects**	*OR (95% CI)*		
*Age*			
15 to 20	Ref	Ref	Ref
20 to 30	0.86 (0.69, 1.08)	1.28 (1.01, 1.61)	1.09 (0.84, 1.43)
30 to 40	0.54 (0.42, 0.69)	1.38 (1.07, 1.77)	1.15 (0.86, 1.53)
40 to 50	0.43 (0.29, 0.62)	1.09 (0.74, 1.6)	0.91 (0.59, 1.41)
*Total # children delivered*	0.64 (0.57, 0.72)	0.85 (0.73, 1)	0.87 (0.73, 1.04)
*Wealth quintile*			
Poorest	Ref	Ref	Ref
Poorer	1.41 (1.32, 1.51)	1.10 (1.02, 1.20)	1.28 (1.15, 1.41)
Middle	1.78 (1.65, 1.92)	1.18 (1.08, 1.29)	1.43 (1.29, 1.59)
Richer	2.50 (2.31, 2.71)	1.36 (1.24, 1.49)	1.69 (1.52, 1.88)
Richest	4.71 (4.22, 5.25)	1.52 (1.36, 1.70)	1.89 (1.67, 2.14)
*Education*			
No education	Ref	Ref	Ref
Primary	1.61 (1.51, 1.72)	1.04 (0.96, 1.12)	1.33 (1.21, 1.45)
Secondary	3.04 (2.75, 3.37)	1.29 (1.16, 1.43)	1.63 (1.44, 1.84)
Higher	7.63 (5.91, 9.84)	2.82 (2.35, 3.39)	1.92 (1.61, 2.28)
*Residence*			
Urban	Ref	Ref	Ref
Rural	0.69 (0.64, 0.75)	1.00 (0.92, 1.08)	0.90 (0.82, 0.98)
*Inaccessibility to nearest facility*	0.24 (0.19, 0.30)	0.74 (0.61, 0.89)	0.58 (0.45, 0.75)
*Age x Total # Children Delivered*			
15 to 20	Ref	Ref	Ref
20 to 30	1.19 (1.05, 1.34)	1.05 (0.90, 1.22)	1.01 (0.84, 1.21)
30 to 40	1.39 (1.23, 1.57)	1.08 (0.93, 1.26)	1.06 (0.88, 1.27)
40 to 50	1.44 (1.27, 1.64)	1.15 (0.98, 1.35)	1.10 (0.92, 1.33)
**Random Effects**	**SBA**	**ANC**	**PNC**
	*Variance (SD)*		
*DHS Region*	0.1923 (0.4385)	0.1422 (0.3770)	0.2248 (0.4741)

Living in rural areas was also associated with decreased odds of obtaining MNH care across outcomes. Specifically, those living in rural areas were 30% less likely to deliver with a skilled birth attendant present (0.69; CI: 0.64–0.75) and 10% less likely to obtain PNC after delivery (0.90; CI: 0.82–0.98) as compared to those living in urban areas. However, while living in rural areas was also associated with decreased odds of receiving 4+ ANC visits, this was not significant (1.00; CI: 0.92–1.08). Lastly, we found age and total number of children delivered were highly correlated, in line with previous studies.[39] Because both age and parity are important indicators in determining service use with sometimes opposing directionality, we included an interaction term in the model to control for the relationship between number of children delivered and respondent’s age. This interaction term improved model fit and was highly significant in explaining skilled birth attendance (p<0.0001), with older women experiencing a greater increase in odds of SBA per child delivered as compared to younger women, and is in line with previous findings.[16] While similar patterns were observed among ANC and PNC outcomes, these effects were not significant.

Finally, an increasing geographic inaccessibility score to the nearest health facility was associated with the greatest reduction in odds of utilising MNH care, particularly among skilled birth attendance. Specifically, for each unit increase in inaccessibility to the nearest health facility, the odds of having a skilled birth attendant present at delivery was reduced by over 75% (0.24; CI: 0.19–0.30), while odds of receiving 4+ ANC visits decreased by nearly 25% (0.74; CI: 0.61–0.89) and 40% for obtaining PNC (0.58; CI: 0.45–0.75). Due to how we calculated inaccessibility scores (as outlined in Methods), these scores do not represent a directly interpretable index of travel time/difficulty, but quantify distance in grid cells to the nearest health facility, scaled by the relative speed of traversing each grid cell type. Overall, these results suggest decreasing accessibility to the nearest health facility significantly deterred utilisation of all maternal health care services.

### High-resolution mapping of MNH care utilisation

**Fig 3:**
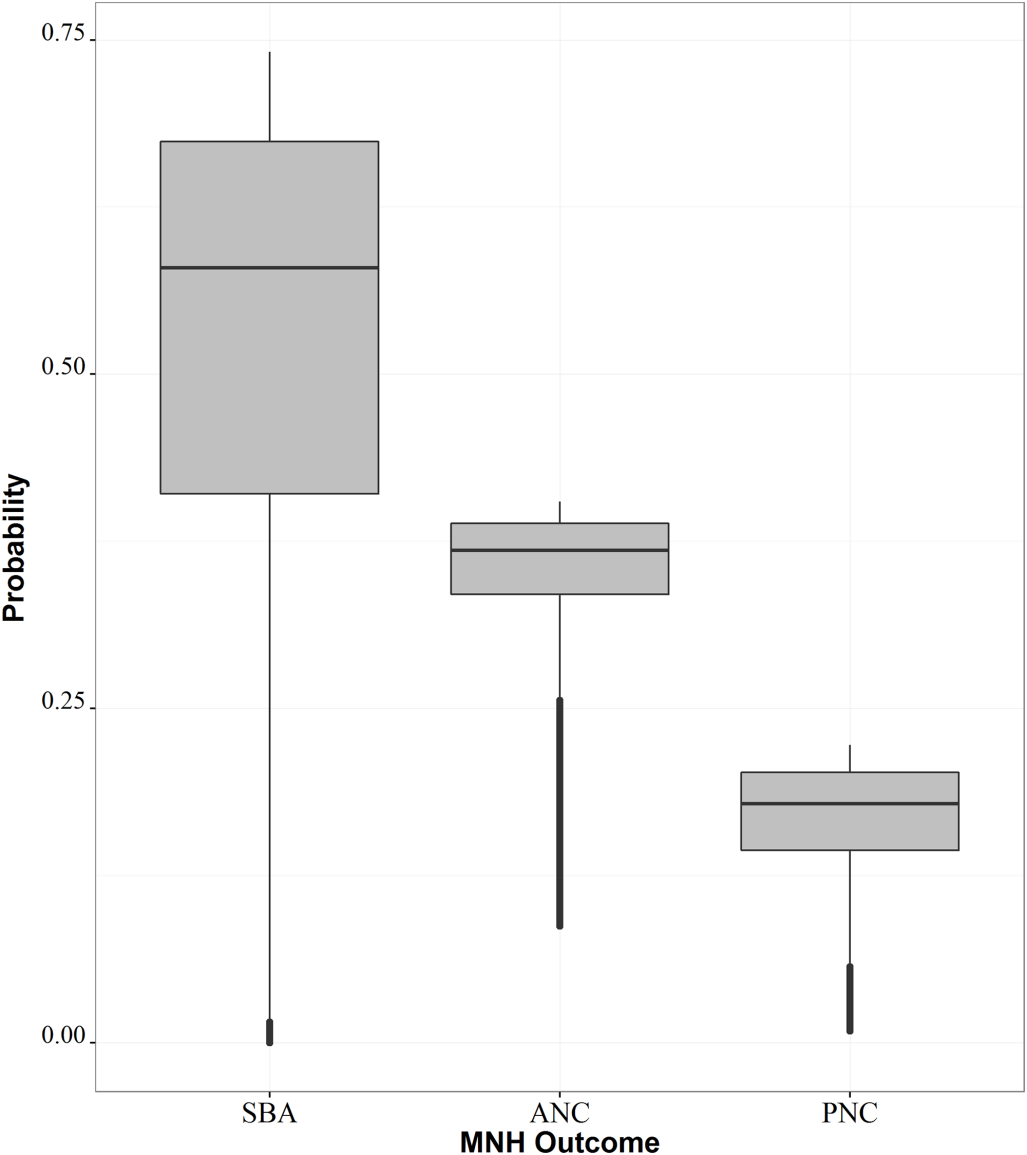
Boxplots of modelled probabilities of skilled birth attendance (SBA), antenatal care (ANC), and postnatal care (PNC).

We applied coefficients from the resulting model of best fit to the previously described accessibility surface to generate high-resolution maps reflecting probability of obtaining MNH care as an emergent effect of accessibility. Fig 3 presents boxplots for these distributions, while S3 Fig presents high-resolution probability surfaces among all MNH outcomes. The probability of having a skilled birth attendant during delivery exhibited the greatest amount of variability across the study region (Fig 3), with an average probability of 58%, ranging between nearly 0% and 75% for a given 300 x 300 m square. The ranges of receiving ANC and PNC were lower than SBA, with a mean probability of 37% (ranging from 9% to 40%) and 18% (ranging from <1% to 22%), respectively.

S3 Fig shows the probability of MNH outcomes at 300 x 300 m, representing spatial heterogeneity in rates of obtaining care as a function of accessibility. As previously discussed, the ranges of probabilities varied widely between outcomes, making direct comparison between probability surfaces difficult due to the varying scales used between subfigures. Instead, this figure shows variations in the emergent spatial patterns resulting from geographic accessibility between outcomes, with the most drastic effect of space observed among SBA, as represented by more contained pockets of higher SBA probability surrounding health facility locations. This spatial effect becomes coarser for ANC and PNC utilisation, with relatively similar patterns observed between the two, despite varying probability distributions (Fig 3).

### Sub-national mapping of MNH care utilisation

**Fig 4:**
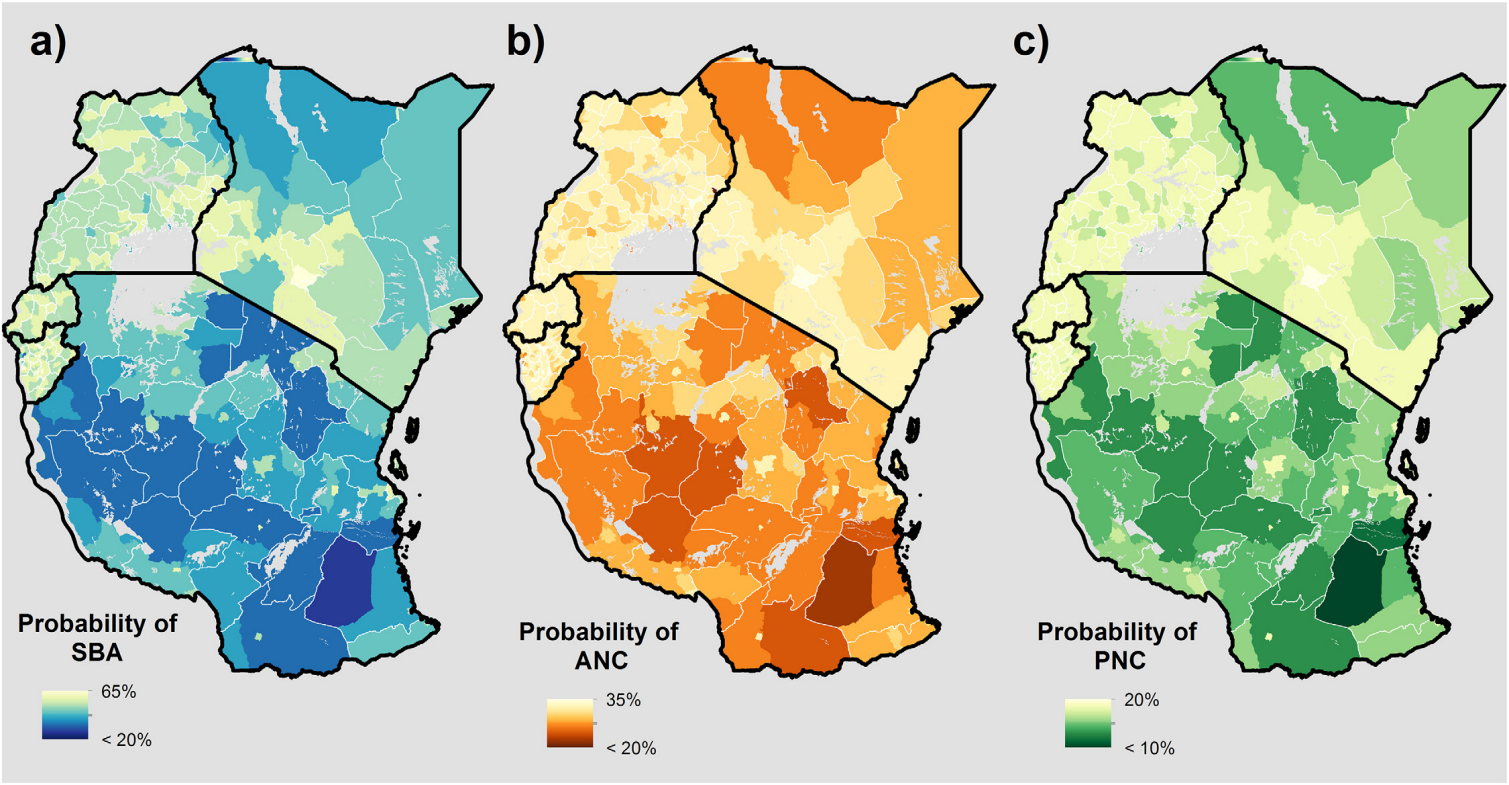
Births adjusted probability maps representing the probability of obtaining MNH care for a given birth at the administrative II unit. **a)** Delivery with a skilled birth attendant (SBA) present, **b)** Four or more antenatal care (ANC) visits at time of delivery, and **c)** Postnatal care (PNC) received within 48 hours of delivery.

To facilitate policy relevance, we aggregated high-resolution probability surfaces to an administratively relevant scale, and adjusted these values using the surface of births per 300 m grid square, as previously described above. Fig 4 therefore represents the probability of: a) having a skilled attendant present during delivery, b) obtaining 4+ ANC visits by time of delivery, and c) the mother receiving PNC within 48 hours of delivery, respectively. Because we adjusted probabilities to reflect actual births at-risk, these maps represent the mean probability of obtaining MNH for a given birth within each geographic unit, accounting for where live births are most likely to occur.

Similar to the high-resolution surfaces, the observed ranges of probabilities varied between countries, with Kenya and Tanzania predominantly driving the scale of heterogeneity across outcomes. Regionally, the lowest probabilities of receiving MNH care occurred throughout northern Kenya and central Tanzania across outcomes. These probabilities generally tended to be higher in urban versus rural districts across countries and outcomes. Conversely, our model results showed Rwanda and Burundi to have consistently higher probabilities of a given birth receiving MNH care, as compared to Uganda, Tanzania and Kenya. Further, the range of these probabilities between administrative units varied less, suggesting less inequality between districts.

## Discussion

Spatial inequalities in utilisation of MNH care continue to persist among low- and middle-income countries, particularly among skilled birth attendance and antenatal care coverage, and targeting spatial pockets of low utilisation will be critical for future health interventions.[2] A nuanced understanding of how geographic accessibility influences uptake of MNH care at a very fine spatial resolution will be key to identify and reduce these inequalities and alleviate coverage gaps. Here, we have highlighted the emergent spatial patterns of MNH care resulting from geographic accessibility at a high-resolution scale, and presented probabilities of obtaining MNH care for a given birth at the sub-national level. The spatial patterns revealed have important policy implications for informing allocation of future intervention efforts to target the most disadvantaged women and riskiest pregnancies. As these analyses highlight areas of low geographic accessibility, the presented results could help target not only health-related resources, but also future development more generally, including road networks and other transportation-related infrastructure.

Overall, we found that disparities exist in obtaining MNH care across wealth, education, and levels of rurality, and that decreased accessibility to the nearest health facility resulted in the widest disparities in obtaining care across the spectrum of pregnancy (Table 1). These findings are in line with previous studies, further establishing that barriers in deciding how, when, and even whether to seek care are greatest for uneducated, remote women in poverty.[9,11,40] Regionally, we found that Kenya and Tanzania had the strongest patterns of spatial heterogeneity in the observed outcomes and generally lower probabilities of obtaining all types of care, with the lowest probabilities observed throughout rural districts in northern Kenya and central Tanzania. Conversely, we found Rwanda and Burundi to have generally higher probabilities of obtaining care, as compared to Tanzania, Kenya, and Uganda. This trend could be due in part to the relative density of facilities available, while more remote areas of Kenya and Tanzania had comparatively less facilities and primary or secondary road networks. This pattern also occurred sub-nationally, as we observed higher probabilities of obtaining care in urban versus rural districts, indicating infrastructure density is important in increasing MNH care coverage.

These findings suggest the allocation of funds focused on supporting increased infrastructure, such as buildings, equipment, and health workers, should appropriately reflect the demography and epidemiology of the area. These analyses could help to direct the flow of such resources, by highlighting areas and populations where care utilisation rates are lowest. While such infrastructure can improve geographic impedance and therefore accessibility, placement of primary health facilities offering comprehensive obstetric care or maternity waiting homes in these particularly resource-poor areas will ultimately be critical in reducing adverse MNH outcomes such as maternal and neonatal mortality.[41] Specifically, our results highlight northern Turkana, Samburu, Marsabit, Wajir, and Mandera districts in Kenya and central Rufiji and Liwale districts in Tanzania as key targets for increased coverage of facilities offering maternity care and construction of better road networks and transportation services. However, because these analyses did not capture information on use of lower tier facilities, referral networks, or community health workers linked to larger health facilities, MNH care utilisation in these areas may artificially be low. Therefore, to promote a more accurate representation of facility coverage in these areas, future data collection efforts should focus on these areas of low geographic accessibility, capturing services provided and quality of services.

Future research should explicitly compare health systems throughout these countries, particularly in Rwanda and Burundi, to understand why MNH care performance is comparatively better in some countries as opposed to others. In particular, previous studies have noted that while MNH outcomes such as neonatal mortality are positively correlated with distance to closest health facility, health facility placement may already be saturated in countries that have prioritised improving facility coverage.[13] In such cases, development of existing infrastructure, including continued support and education opportunities for community health workers, improved road networks and public transportation, and increased availability of comprehensive obstetric facilities or maternity waiting homes may ultimately prove effective in alleviating geographic accessibility as a barrier to accessing maternal health services. Finally, to promote a more nuanced and accurate understanding of geographic accessibility, future data collection efforts should aim to capture information on specific health facility used during pregnancy and birth, mode of transportation used to access these facilities, as well as self-reported travel time to these facilities.

These analyses are subject to several limitations. Firstly, by excluding dispensaries from the analyses, it is possible that we incorrectly identify some women as having more difficulty in obtaining MNH care than is actually the case. However, we chose to be overly conservative in our modelling efforts by excluding these facilities, to avoid incorrectly assuming that a woman would have access to MNH services that are not actually there. Secondly, we use self-reported MNH outcomes and definitions, which may vary from country to country and may be subject to recall bias. This limitation explicitly impacts skilled birth attendance analyses, as some women may be unaware of what type of attendant was present at delivery, and countries may further define “skilled attendants” differently. While we limited our analyses of skilled attendance only to doctors, nurses and midwives, it is possible countries may define nurses and midwives variably. Future studies should ensure that definitions can be standardized on a multi-national level, and examine impacts of these definitions on model results. Thirdly, we assumed in our analyses that women would obtain care by travelling to the nearest health facility to their house. This oftentimes, however, does not occur in reality and is influenced by a host of individual and systematic factors, which may include, among others, the use of multiple facilities to obtain care via referral networks, the quality of care provided at the facility, and individual perception of the facility.[9] Future work should more explicitly examine respondents’ actual use of health facilities, and explore key factors explaining where a woman decides to obtain care. Finally, our analyses were limited temporally by survey availability. Specifically, with data ranging over a 4 year span throughout the study countries, and gathering information on births in the previous 5 years, it is possible, that these results may no longer reflect the current state of maternal and newborn health in some countries. Future analyses should examine change in utilisation of MNH care over time, and use more recent datasets where available.

## Conclusions

Inequalities in obtaining MNH care continue to persist, despite progress in increasing coverage and availability amongst the most vulnerable subgroups in the world. Spatial disaggregation of MNH data and a nuanced understanding of the geographic processes driving these disparities will be critical to continue this progress and accelerate achievement of SDG goals to reduce health disparities among all. Here, we spatially modelled the probability of several MNH outcomes at both high-resolution and policy relevant scales to highlight spatial patterns in accessibility and sub-national inequalities in MNH care utilisation throughout central East Africa. We found that disparities exist across the socioeconomic spectrum, with the widest disparities observed in geographic accessibility to health facilities, particularly among skilled birth attendance. The results of these analyses demonstrate how spatial approaches can be used to measure and identify spatial pockets of historically overlooked inequalities, thereby strategically informing policy efforts and promoting evidence-based decision making. These findings are particularly pertinent to the East African Community in its efforts to accelerate progress in women’s, children’s and adolescent’s health and equity within the framework of the Sustainable Development Goals.

## Supporting Information

S1 Fig Geo-located DHS clusters (N = 3,311) by number of DHS respondents (N = 36,178) and urban versus rural location in five East African countries. (TIF)

S2 Fig Estimated live births in five East African countries. Birth estimates were generated by the WorldPop project (www.worldpop.org) and are shown for the year 2015 at 100 x 100 m resolution.
(TIF)

S3 Fig Modelled probability surfaces representing the spatial effect of accessibility at 300 x 300 m. **a)** Delivery with a skilled birth attendant (SBA) present, **b)** Four or more antenatal care (ANC) visits at time of delivery, and **c)** Postnatal care (PNC) received within 48 hours of delivery.
(TIF)

S4 Fig ROC curves for skilled birth attendance (SBA), antenatal care (ANC), and postnatal care (PNC) models, with associated area under the curve (AUC) metrics. (TIF)

S1 File Model validation details. (DOCX)

S2 File Final health facilities used in analysis (N = 9,314). List includes facility name, owner, and status (all where available), plus latitude, longitude, and ISO codes for study countries.
(CSV)
