# Correction: Equality in Maternal and Newborn Health: Modelling Geographic Disparities in Utilisation of Care in Five East African Countries

**DOI:** 10.1371/journal.pone.0164519

**Published:** 2016-10-06

**Authors:** Corrine W. Ruktanonchai, Nick W. Ruktanonchai, Andrea Nove, Sofia Lopes, Carla Pezzulo, Claudio Bosco, Victor A. Alegana, Clara R. Burgert, Rogers Ayiko, Andrew SEK Charles, Nkurunziza Lambert, Esther Msechu, Esther Kathini, Zoë Matthews, Andrew J. Tatem

The following information is missing from the Funding section: This work was funded through the Norwegian Development Agency (NORAD), grant number RAF-2946 RAF-12/0094, in collaboration with ICS Integrare.

## References

[1] Ruktanonchai CW, Ruktanonchai NW, Nove A, Lopes S, Pezzulo C, Bosco C, et al (2016) Equality in Maternal and Newborn Health: Modelling Geographic Disparities in Utilisation of Care in Five East African Countries. PLoS ONE 11(8): e0162006 doi: 10.1371/journal.pone.0162006 27561009 10.1371/journal.pone.0162006 PMC4999282

