# Supplementary figures and images for: Equality in Maternal and Newborn Health: Modelling Geographic Disparities in Utilisation of Care in Five East African Countries
Source: PLoS One. 2016 Aug 25;11(8):e0162006. doi: 10.1371/journal.pone.0162006 (PMC4999282; doi:10.1371/journal.pone.0162006)

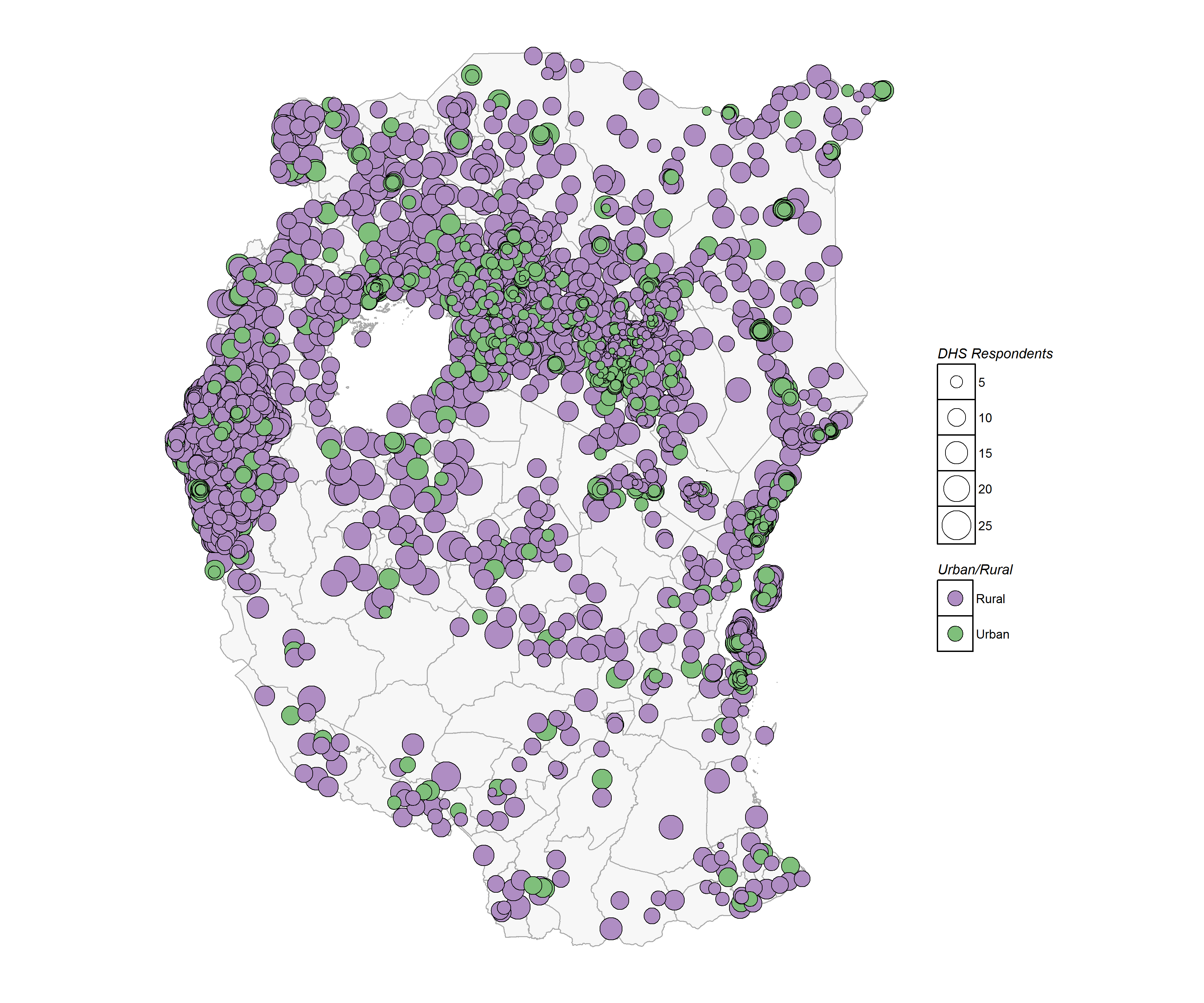

Supplement: S1 Fig — (TIF) [file pone.0162006.s001.tif]

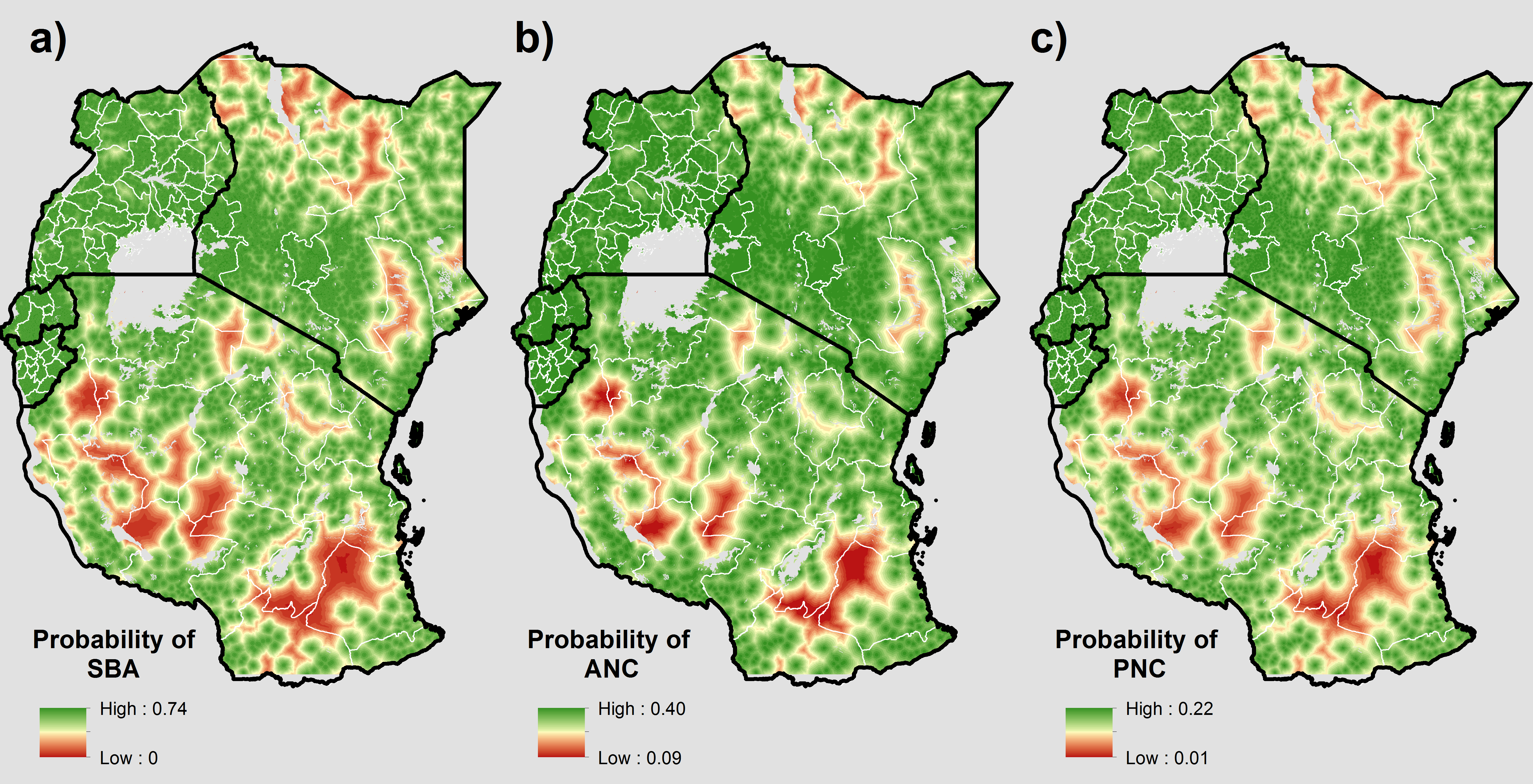

Supplement: S3 Fig — a) Delivery with a skilled birth attendant (SBA) present, b) Four or more antenatal care (ANC) visits at time of delivery, and c) Postnatal care (PNC) received within 48 hours of delivery. (TIF) [file pone.0162006.s003.tif]

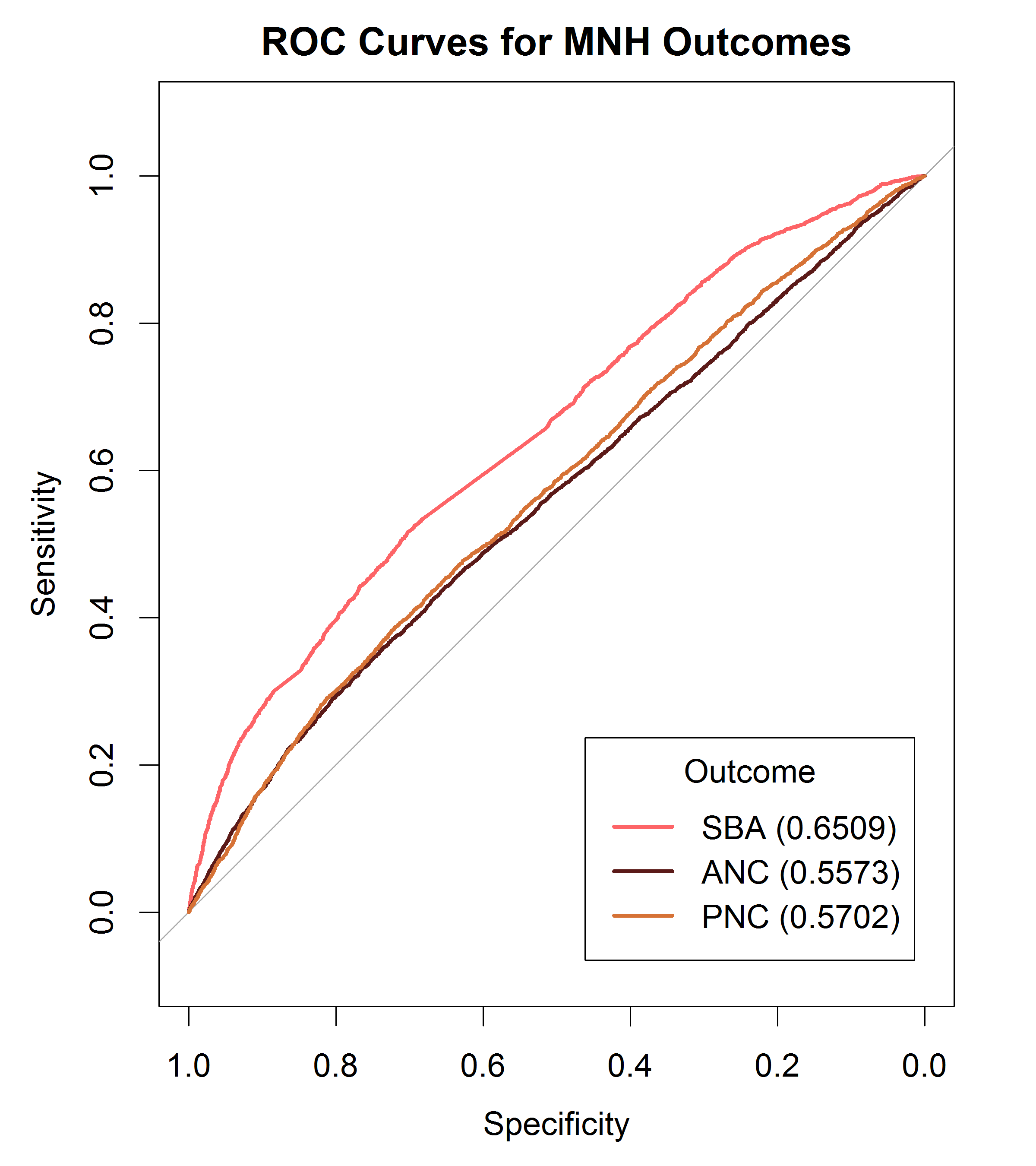

Supplement: S4 Fig — (TIF) [file pone.0162006.s004.tif]
