# Supplementary material for: Equality in Maternal and Newborn Health: Modelling Geographic Disparities in Utilisation of Care in Five East African Countries
Source: PLoS One. 2016 Aug 25;11(8):e0162006. doi: 10.1371/journal.pone.0162006 (PMC4999282; doi:10.1371/journal.pone.0162006)
Supplement: S1 File — (DOCX) [file pone.0162006.s005.docx]

**Model Validation**

To test the ability of our regression models to correctly predict utilisation of skilled birth attendance and antenatal/postnatal care, we performed a receiver operating characteristics (ROC) analysis. This method has been widely used within epidemiological fields to evaluate diagnostic tools, with recent applications in validating regression models within a spatial context.[1,2] Briefly, the area under the ROC curve (AUC-ROC) represents the discriminatory power of the model in predicting true prevalence of a threshold, representing a trade-off between sensitivity and specificity.[3] Higher AUC values represent increasing discriminatory ability of the model, or the ability to correctly classify predicted observations as receiving or not receiving a given service. S4 Fig outlines the results of the ROC analysis, and provides metrics of the AUC-ROC, with the skilled birth attendance model performing relatively better than the ANC and PNC models. With an AUC value of 0.65, this model is deemed a moderately well-fitting model, and indicates a 65% probability that a woman observed with SBA had a higher estimated probability of SBA than a woman without SBA, for a random pair of women with and without the outcome.[1]

1. Clements ACA, Lwambo NJS, Blair L, Nyandindi U, Kaatano G, Kinung’hi S, et al. Bayesian spatial analysis and disease mapping: tools to enhance planning and implementation of a schistosomiasis control programme in Tanzania. Trop Med Int Health. 2006;11: 490–503. doi:10.1111/j.1365-3156.2006.01594.x

2. Noor AM, Clements AC, Gething PW, Moloney G, Borle M, Shewchuk T, et al. Spatial prediction of Plasmodium falciparum prevalence in Somalia. Malar J. 2008;7: 159. doi:10.1186/1475-2875-7-159

3. Fawcett T. An introduction to ROC analysis. Pattern Recognit Lett. 2006;27: 861–874. doi:10.1016/j.patrec.2005.10.010
